# *De novo* transcriptome assembly and annotation for gene discovery in *Salamandra salamandra* at the larval stage

**DOI:** 10.1038/s41597-023-02217-9

**Published:** 2023-05-27

**Authors:** Pietro Libro, Andrea Chiocchio, Erika De Rysky, Jessica Di Martino, Roberta Bisconti, Tiziana Castrignanò, Daniele Canestrelli

**Affiliations:** Università degli Studi della Tuscia, Dipartimento di Scienze ecologiche e Biologiche, Largo dell’Università snc, 01100 Viterbo, Italy

**Keywords:** Transcriptomics, Data processing, Molecular ecology

## Abstract

Dispersal is a key process in ecology and evolutionary biology, as it shapes biodiversity patterns over space and time. Attitude to disperse is unevenly distributed among individuals within populations, and that individual personality can have pivotal roles in the shaping of this attitude. Here, we assembled and annotated the first *de novo* transcriptome of the head tissues of *Salamandra salamandra* from individuals, representative of distinct behavioral profiles. We obtained 1,153,432,918 reads, which were successfully assembled and annotated. The high-quality of the assembly was confirmed by three assembly validators. The alignment of contigs against the *de novo* transcriptome led to a mapping percentage higher than 94%. The homology annotation with DIAMOND led to 153,048 (blastx) and 95,942 (blastp) shared contigs, annotated on NR, Swiss-Prot and TrEMBL. The domain and site protein prediction led to 9850 GO-annotated contigs. This *de novo* transcriptome represents reliable reference for comparative gene expression studies between alternative behavioral types, for comparative gene expression studies within Salamandra, and for whole transcriptome and proteome studies in amphibians.

## Background & Summary

Dispersal is a key process in ecology and evolutionary biology, as it contributes to shaping the spatial patterns of biodiversity and their variation over space and time^1–3^. Recently, growing evidence has been shown the importance of the role of animal personality in affecting dispersal processes^4–6^. In particular, some personality traits have been linked to a higher – or lower – individual attitude to disperse and/or to survive in recently colonized environments. Therefore, behavioral polymorphisms in dispersal-related traits can affect the spatial patterns of intraspecific genetic variation and can play an active role in driving eco-evolutionary pathways. Recent advances in the study of the heritability of animal personality indicate the substantial contribution of additive genetic variance to behavioral trait variation^7^. However, we still miss a thorough understanding of the genetic underpinnings of behavioral polymorphisms related to dispersal.

Amphibians provide an intriguing opportunity to study dispersal ecology. By moving from aquatic to terrestrial habitats, amphibians face with substantial niche shifts at metamorphosis, which is linked to ontogenetic changes in morphology, physiology, and behavior^8^. Such a dramatic change in habitat features also requires substantial changes in dispersal-related traits. Noteworthy, conditions experienced during the aquatic larval stage (e.g., water temperature, predation rates, and conspecific density) can shape post-metamorphic phenotypes and thus influence dispersal process after metamorphosis^9^. This results in complex carryover effects on dispersal-related traits that add complexity to identifying patterns and mechanisms of dispersal compared to taxa with simple life cycles^10^.

In this study, we aim to contribute to the understanding of the genetic basis of behavioral polymorphisms related to dispersal in amphibians, by assembling the *de novo* transcriptome of the larval stage of the fire salamander *Salamandra salamandra*, a species showing marked polymorphism in dispersal-related personality traits. The fire salamander is a stream-breeding amphibian widespread in the western Palearctic region, characterized by rather low dispersal attitudes^11^. A recent study on the inter-individual behavioral variation in the Italian population of the fire salamander, showed marked polymorphisms in dispersal-related behavioral profiles of larvae and juveniles^12^. In particular, two distinct profiles have been identified within populations: a less active and less exploratory behavioral profile, and a more active and exploratory behavioral profile. Interestingly, this polymorphism has been associated with a marked differentiation between two co-occurring mitochondrial DNA lineages, not mirrored at the level of the nuclear genome^12^.

Here, we focused transcriptome analyses on tissues extracted from the cephalic region, as the brain is a target tissue for investigating the genetic background of behavior^13,14^. In fact, there is evidence showing that brain gene expression patterns can reflect behavioral state in response to environmental stimuli^15,16^. Thus, transcriptome analyses of the brain can contribute to reveal the genetic architecture of animal personalities^17–19^. The transcriptome presented here has been validated and annotated, in order to provide a reference for further analysis. Furthermore, because of its large size, the *S. salamandra* genome is not still available, and thus this transcriptome will join the other transcriptome data for this species^20–22^ to provide a valuable genomic resource for further ecological and behavioral studies.

## Methods

### Experimental design

We collected salamander larvae from a population in Central Italy showing behavioral polymorphism^12^ (Picentini Mountains: 40°48′ N, 14°53′ E). Details about sampling, housing and behavioral essays are described in Chiocchio *et al*.^12^. We selected 10 larvae of fire salamander representative of two distinct behavioral profiles, i.e., slow, less active and less exploratory behavior *vs* fast, more active and more exploratory behavior (thereafter referred as “slow” and “fast”, respectively; see Table 1). For each individual, the cephalic region was dissected and immediately stored in RNAprotect Tissue Reagent (Qiagen) until RNA extraction. All procedures followed the relevant guidelines and regulations for welfare and were approved by the Italian Ministry of Environment (permit number: 0008275.20-04-2018), the Institute for Environmental Protection and Research (#23501, 23-03-2018) and “Regione Campania” (#0203190, 27-03-2018). Permission to temporarily house amphibians at the University facilities was granted by the local health and veterinary authority (A.S.L. Tarquinia, license 050VT427).

**Table 1:** Summary of the 10 libraries deposited in the ENA (European Nucleotide Archive, Study Accession Id PRJEB51202), in terms of number of raw and trimmed reads per sample.

Sample code	Phenotype	Run ID	Raw sequences	Filtered sequences (% of the total reads)	% Trimmed reads
R530	slow	ERR8963532	148,383,674	143,289,768	96.57
R531	fast	ERR8971203	113,170,212	108,848,306	96.18
R538	slow	ERR8971371	131,207,312	126,584,688	96.48
R541	fast	ERR8962162	10,147,648	97,683,726	96.27
R547	fast	ERR8971605	116,726,758	112,808,264	96.64
R560	slow	ERR8971694	123,732,768	119,419,898	96.51
R564	slow	ERR8971822	99,188,392	95,748,700	96.53
R565	fast	ERR8972251	98,856,326	95,578,524	96.68
R572	fast	ERR8972978	117,839,158	113,636,400	96.43
R573	slow	ERR8974269	102,857,670	98,754,492	96.02

### Dataset generation

RNA extractions were performed using the RNeasy Plus Kit (Quiagen) on approximately 60 mg of tissue, according to the manufacturer’ instructions. RNA quality and concentration were assessed by means of either a spectrophotometer and a Bioanalyzer (Agilent Cary60 UV-vis and Agilent 2100, respectively - Agilent Technologies). From each individual, we were able to extract 7.2 to 22.3 ug of total RNA. RNA integrity numbers (i.e., RIN) ranged from 8.5 to 9.

Library preparation and RNA sequencing were performed by NOVOGENE (UK) COMPANY LIMITED using the Illumina NovaSeq platform. Library construction was carried out using the NEBNext® Ultra ™ RNA Library Prep Kit for Illumina®, following manufacturer instructions. Briefly, after quality control, the mRNA present in the total RNA sample was isolated with magnetic beads of oligos d(T)25 (*i.e*., polyA-tailed mRNA enrichment). Subsequently, mRNA was randomly fragmented and cDNA synthesis proceeded by random hexamers and the reverse transcriptase enzyme. Once the synthesis of the first chain was finished, the second chain was synthesized by means of the Nick translation method, with the addition of dNTPs, RNase H, polymerase I of *E. coli*. The resulting products went through purification, repair, A-tailing and adapter ligation. Fragments of the appropriate size were then enriched by PCR, the indexed P5 and P7 primers were introduced, and the final products were purified. The Illumina NovaSeq 6000 sequencing system was used to sequence the libraries, through a paired-end 150 bp (PE150) strategy. We obtained on average 52.7 million reads for each library. The sequencing data are available at the NCBI Sequence Read Archive (see Table 1).

### Pre-assembly processing stage

**Fig. 1:**
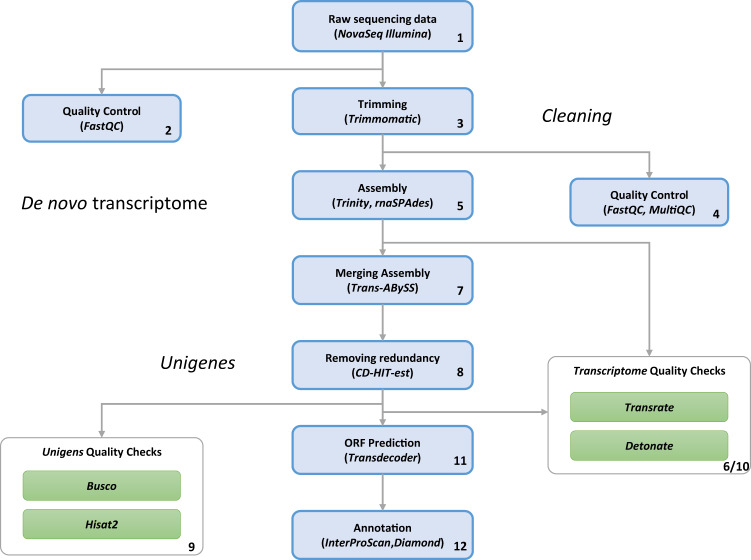
Workflow of the bioinformatic pipeline, from raw data to annotated scripts, for the *de novo* transcriptome assembly of *S. salamandra*. Each step was progressively numbered.

Data from the brains of larvae were derived from ten independent samples and processed for bulk transcriptome sequencing. The workflow of the bioinformatic pipelines is shown in Fig. 1. All the described bioinformatics analyses were performed on the high-performance computing systems provided by ELIXIR-IT HPC@CINECA^23–25^.

We obtained 1,153,432,918 pairs of reads. The quality of the raw reads was assessed with FastQC 0.11.5 (http://www.bioinformatics.bbsrc.ac.uk/projects/fastqc). The quality results were aggregated across all samples into a single report with MultiQC^26^ v. 1.9. Raw reads were then analyzed through a quality trimming step with Trimmomatic^27^ v. 0.39 (options SLIDINGWINDOW: 4: 15, MINLEN: 36, HEADCROP: 13), in order to remove low quality bases and adapter sequences. Unpaired reads were discarded. After the cleaning step and removal of low-quality reads, a total amount of 1,112,352,766 clean reads were maintained for building the *de novo* transcriptome assembly (i.e., 96% of raw reads, Table 1).

### *De novo* transcriptome assembly

Because there is no reference genome for *S. salamandra*, we performed a *de novo* transcriptome assembly following the workflow of the bioinformatic pipeline described in Fig. 1. In order to construct an optimized *de novo* transcriptome, avoiding chimeric transcripts and improving the reliability of the final assembly, we adopted the strategy to launch two *de novo* assembly tools both based on the building of de bruijn graphs, particularly suitable for eukaryotic organisms. The two assemblers were Trinity^28^, v. 2.11.0, and SPAdes^29^, v. 3.11.1, used in rnaSPAdes mode. rnaSPAdes was then applied with the default parameters (k-mer size equal to 73) to provide the assembly result. rnaSPAdes run generated as output a total of 1,094,271 transcripts (Table 2). On the other hand, also Trinity was launched applying the default parameters (kmer size equal to 25) to provide the assembly result. At this stage, a total of 1,207,872 transcripts were generated as output of Trinity run (Table 2).

**Table 2:** Statistics on rnaSPAdes and Trinity output and the result after CD-HIT evaluated with the two assembly validators.

	Trinity	rnaSPAdes	CD-HIT-est (Unigens)
**Basic parameters**			
Total transcripts	1,207,872	1,094,271	1,146,571
N50	1742	1979	1529
GC content (%)	45.0	45.0	44.7
**TransRate v.1.0.3**			
Transrate Assembly Score	0.05	0.06	0.06
Transrate Optimal Score	0.1	0.09	0.1
Transrate Optimal Cutoff	0.01	0.01	0.01
good contigs	947,064	974,586	972,431
p good contigs	0.8	0.9	0.9
**DETONATE v.1.9**			
Score	−4.8e10	−3.9e10	−4.3e10
BIC_penalty	−1.2e7	−1.1e7	−1.1e7
Prior_score_on_contig_lengths_(f_function_canceled)	−2.5e6	−2.3e6	−2.1e6
Prior_score_on_contig_sequences	−1.3e9	−1.1e9	−1.1e9
Data_likelihood_in_log_space_without_correction	−4.6e10	−3.8e10	−4.2e10
Correction_term_(f_function_canceled)	−5.0e6	−4.9e	−5.1e6

After the assembly step, the two output results were merged using Trans-AbySS^30^ v. 2.0.1, with the merging function.

### Assessing assembly quality

Two validation steps were applied to the assembly results: one after step 5, to evaluate the preliminary assembly, and one after step 8, to assess the quality of the final, non-redundant, assembly output. Two different tools were used for this task: TransRate^31^, v. 1.0.3, and DETONATE^32^, v. 1.11. These tools generate several metrics that serve as a guide to evaluate error sources in the assembly process and provide evidence about the quality of the assembled transcriptome. In Table 2 we reported the assessment analyses of a) the assembly output of Trinity, b) the assembly output of rnaSPAdes, c) the final assembly output, i.e., the merged assembly with removed redundancies.

**Fig. 2:**
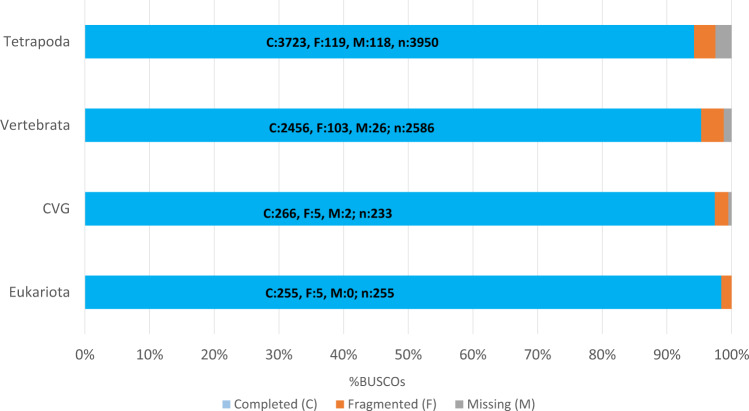
BUSCO assessment results.

The quality of the final assembly (output from step 8) was further evaluated through the assessment procedure implemented in BUSCO^33^ (Benchmarking Universal Single-Copy Orthologs) v. 5.4.4. It provides a quantitative measure of transcriptome quality and completeness, based on evolutionarily informed expectations of gene content from the near-universal, ultra-conserved proteins databases. We analyzed the gene content by launching BUSCO, on four databases of ortholog genes: CVG (Core Vertebrate Genes), Tetrapoda, Vertebrata and Eukariota databases. In Table 3 we reported transcriptome completeness in BUSCO. Moreover, Fig. 2 shows completed, fragmented and missing genes mapped from the four databases. It is worth noting that we found a high percentage of completed genes on Tetrapoda and Vertebrata databases, confirming the good quality of our assembly.

**Table 3:** The BUSCO (v. 5) validation, though the gVolante web server^49^ was applied to four databases: Tetrapoda, Vertebrata, CVG (Core Vertebrate Genes) and Eukariota.

Busco Category	Tetrapoda Database	Vertebrata Database	CVG (Core Vertebrate Genes)	Eukaryota Database
Complete BUSCOs (C)	3723 (94.2%)	2456.7 (95.3%)	226 (97.4%)	255 (98.4%)
Complete and single-copy BUSCOs (S)	1975 (50.0%)	1318.9 (50.7%)	111.84 (48.5%)	96.9 (37.6%)
Complete and duplicated BUSCOs (D)	1739 (44.2%)	1163.7 (44.6%)	114.2 (48.9%)	155.5 (60.8%)
Fragmented BUSCOs (F)	119 (3.3%)	103.4 (3.5%)	4.6 (2.1%)	5.1 (1.6%)
Missing BUSCOs (M)	118.5 (2.5%)	25.9 (1.2%)	2 (0.5%)	0.0%
Total BUSCO groups searched	3950	2586	233	255

**Fig. 3:**
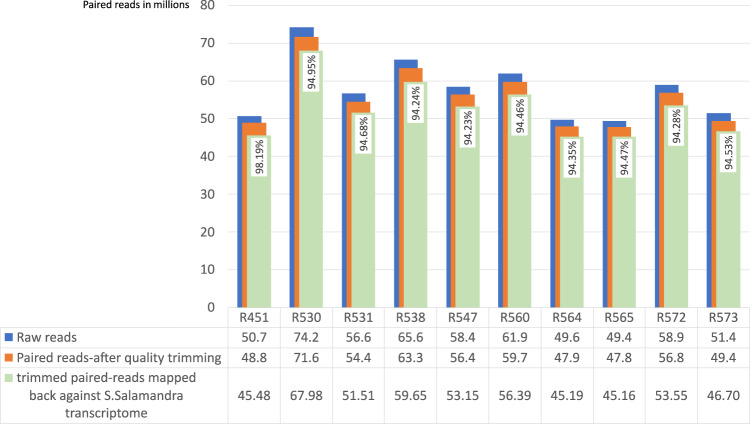
For each sample we have in blue the representation of total paired-reads, in orange the total paired-reads after the adapter removal and quality trimming and in azure we have the trimmed paired-reads mapped mapped-back against the *Salamandra salamandra* assembled *de novo* transcriptome.

Another kind of quality assessment evaluation was introduced by performing mapping the trimmed raw data against the obtained final *de novo* transcriptome assembly. The software tool used for mapping was HISAT2^34^ v. 2.1.0, one of the fastest and most widely used open-source gene alignment resources. The mapping results are shown in Fig. 3. As can be noticed, the results are always higher than 94%, confirming the good quality of the assembly.

### Generation of the full-length transcriptomes

After the validation step (“Transcriptome Quality Check” in Fig. 1), the merged assembly was the input for CD-HIT-est^35^ program, v. 4.8.1, a hierarchical clustering tool used to avoid redundant transcripts and fragmented assemblies common in the process of the *de novo* assembly, providing unique genes. CD-HIT-est was run with default parameters, corresponding to a similarity of 95%. Subsequently, a second validation step (“Unigenes Quality Checks” in Fig. 1) was launched on the CD-HIT-est output file. The results are shown in Table 2. Then, the CD-HIT-est output file was run on TransDecoder^36,37^ v5.7.0, the current standard tool that identifies long open read frames (ORFs) in assembled transcripts, using the default settings. TransDecoder by default performs ORFs prediction on both strands of assembled transcripts regardless of the sequenced library, i.e., without considering the specific sequencing library used to generate the transcriptomic data. It also ranks ORFs based on their completeness, by looks for any length of AA codons upstream of a start codon (M) without a stop codon, to determine if the 5′ end is incomplete. We adopted the “Longest ORF” rule and selected the longest 5′ AUG (relative to the in-frame stop codon) as the translation start site.

### Transcriptome annotation

**Fig. 4:**
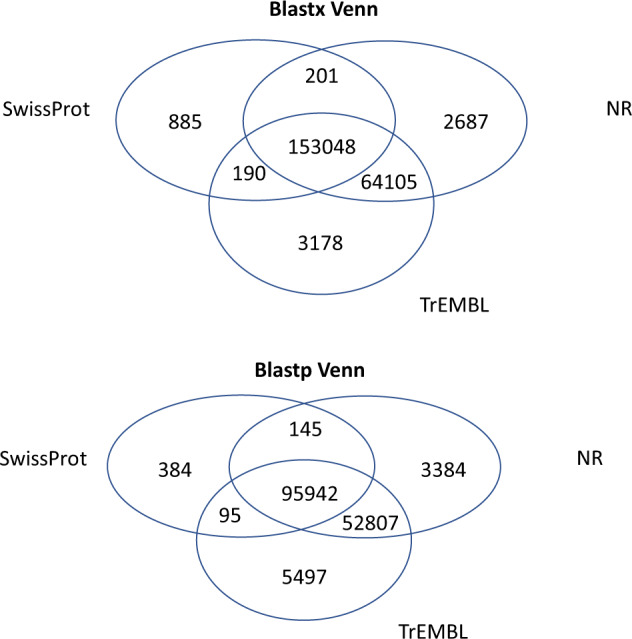
Venn diagrams for the number of contigs annotated with DIAMOND (blastx and blastp functions) against the three databases: NCBI nr, Swiss-Prot, TREMBL.

We employed different annotations for all further analysis. Contigs were aligned with DIAMOND^38^ on NCBI nr, Swiss-Prot and TrEMBL to retrieve corresponding best annotations. An annotation matrix was then generated by selecting the best hit for each database. We applied DIAMOND-fast setting DIAMOND blastx -t 48 -k 250 -min-score 40, and DIAMOND-sensitive, setting DIAMOND blastx -t 48 -k 250 -sensitive -min-score 40. Results from the analysis in DIAMOND are resumed in Table 4. Overview of data files and data sets produced in this study, with information on data repository and accession numbers, are summarized in Table 5. A Venn diagram was created to show the redundancy of the annotations in different databases; the diagrams were constructed for both DIAMOND blastx and DIAMOND blastp (Fig. 4) and showed 153,048 (blastx) and 95,942 (blastp) shared unigenes, i.e., annotated from the three databases. In a further step, contigs were processed with InterProScan^39^, to predict protein signatures. The InterPro database^40^ integrates predictive models or ‘signatures’ representing protein domains, families, and functional sites from multiple, diverse source databases: Gene3D, PANTHER, Pfam, PIRSF, PRINTS, ProDom, PROSITE, SMART, SUPERFAMILY and TIGRFAMs. We scanned the Interpro database using InterProScan (the software package that allows sequences to be scanned against InterPro’s member database signatures) we got 56179 unigenes annotated among which 9850 were GO-annotated and 2311 KEGG-annotated.

**Table 4:** Summary of annotations on different databases.

Annotation statistics	
**Number of blastx results**	
NCBI nr	220,041 (49.86%)
Swiss-Prot	154,324 (34.97%)
TrEMBL	220,521 (49.97%)
**Number of blastp results**	
NCBI nr	152,278 (34.50%)
Swiss-Prot	96,566 (21.88%)
TrEMBL	154,341 (34.97%)

**Table 5:** Overview of data files and data sets produced in this study, with information on data repository.

Label	Name of data file/data set	File types	Data repository and identifier (DOI or accession number)
Data file 1	*S. salamandra* Trinity de novo transcriptome assembly	Fasta file (.fa)	10.6084/m9.figshare.22341469
Data file 2	*S. salamandra* rnaSPAdes de novo transcriptome assembly	Fasta file (.fa)	10.6084/m9.figshare.22341526
Data file 3	*S. salamandra* merged assembly	Fasta file (.fa)	10.6084/m9.figshare.22341439
Data file 4	S. salamandra unigenes	Fasta file (.fa)	10.6084/m9.figshare.22341541
Data file 5	*S. salamandra* Open Reading Frames (ORFs) prediction	Fasta file (.fa)	10.6084/m9.figshare.22341550
Data file 6	*S. salamandra* homology annotation (blastx), NR	Text file (.tsv)	10.6084/m9.figshare.22341577
Data file 7	*S. salamandra* homology annotation (blastx), Swiss-Prot	Text file (.tsv)	10.6084/m9.figshare.22341589
Data file 8	*S. salamandra* homology annotation (blastx), TrEMBL	Text file (.tsv)	10.6084/m9.figshare.22341673
Data file 9	*S. salamandra* homology annotation (blastp), NR	Text file (.tsv)	10.6084/m9.figshare.22341595
Data file 10	*S. salamandra* homology annotation (blastp), Swiss-Prot	Text file (.tsv)	10.6084/m9.figshare.22341679
Data file 11	*S. salamandra* homology annotation (blastp), TrEMBL	Text file (.tsv)	10.6084/m9.figshare.22341694
Data file 12	*S. salamandra* functional annotation InterProScan results	Text file (.txt)	10.6084/m9.figshare.22341715
Data file 13	*P. waltl* transcriptome	Fasta file (.fa)	10.6084/m9.figshare.22341739
Data file 14	*P. waltl* Open Reading Frames (ORFs) prediction	Fasta file (.fa)	10.6084/m9.figshare.22680325
Data file 15	*S. salamandra* CDSs vs *P. waltl* ORFs (by blastx)	Text file (.tsv)	10.6084/m9.figshare.22680244
Data file 16	*P. waltl* CDSs vs *S. salamandra* ORFs (by blastx)	Text file (.tsv)	10.6084/m9.figshare.22680259
Image file 1	*S. salamandra* MultiQC quality assessment	PDF file (.pdf)	10.6084/m9.figshare.22718221

### Comparison with *Pleurodeles waltl* open reading frames

We used the reference genome and the corresponding genome annotation of *Pleurodeles waltl* (the *P. waltl*’s gene model)^41^ to (1) extract the transcriptome of *P. waltl*, (2) predict the corresponding ORFs, (3) map the predicted ORFs of *S. salamandra* versus the predicted ORFs of *P. waltl* and vice versa. The point 3 was implemented to assess the similarity rate between our *de novo* transcriptome of *S. Salamandra* and the transcriptome of *P. waltl*.

As first step we downloaded the genome and annotation files of *P. waltl*^42^, subsequently we used the program GffRead^43^, an open-source program to manipulate GFF and GTF format files, to extract the transcriptome of *P. waltl*. As input files for GffRead, we used the annotation file, aPleWal.anno.v2.20220926.gff3, and the assembled genome, aPleWall.pri.20220803.fasta.gz, which is 20 GB in size. As outcome of the GffRead execution, we obtained the transcriptome of *P. waltl* (the file P_Waltl_transcripts.fa was uploaded on figshare^44^). By TransDecoder we predicted the ORFs of *P. waltl* and, used them to create a blast database. We ran the DIAMOND program (blastx function)^45^ to compare the ORFs of *S. salamandra* (query sequences in multi-fasta format) with the indexed blast database of ORF sequences of *P. waltl*. Of the 441,339 CDS-ORF sequences in the *S. salamandra*, only 290,095 mapped the ORFs of *P. waltl*, representing 65.7% of the total sequences of the *S. salamandra*. Similarly, the CDS-ORFs of *P. waltl* (1180470) were mapped against the ORFs of *S. Salamandra* showing similarity for 792563 of them (67.1%). The blastx output files of both blastx runs, named respectively salamandra_unigenes_vs_pleurodeles_orf_blastx.tsv and pleurodeles_vs_salamandra_unigenes_orf_blastx.tsv, were also uploaded on figshare^44^. The protocol above described provides the comparison of ORFs of *S. salamandra* and *P. waltl* and shows the level of genomic similarity between the two species.

## Data Records

All the raw data generated in this project were deposited in the European Nucleotide Archive (ENA) database under study identification number PRJEB51202^46^. The *de novo* transcriptome assembly resource is available on both the ENA archive, HBZU010000000^47^, and the SRA archive on NCBI HBZU000000000.1^48^. Datasets containing all files produced in this transcriptome assembly and annotation pipeline (Trinity and rnaSPAdes transcriptome assemblies, unigenes, and functional annotation files) were also deposited on figshare archive^44^ (links to pipeline outcomes are listed in Table 5).

## Technical Validation

### Quality of the raw reads and assembly validation

The overall data quality was assessed using FastQC for all samples before and after trimming. Among the FastQC results, the mean quality scores at each base position were higher than 35 (see “Image file 1” in Table 5). Validation of the transcriptome assembly was performed using three validator tools: BUSCO, DETONATE, and TransRate. The results from DETONATE and TransRate validation steps are shown in Table 2, which includes the scores obtained from the execution of the two analysis tools. BUSCO analysis was performed on four databases: Tetrapoda, Vertebrata, CVG, and Eukariota. The details of BUSCO are listed in Table 3, and some of them are plotted, like a histogram, in Fig. 2. A further validation assessment was performed by mapping the trimmed reads against the de novo assembled transcriptome of S. salamandra. The HISAT2 results showed an even higher percentage of 94% (Fig. 3), confirming the very good quality of the assembly. The final transcriptome (unigenes) obtained after CD-HIT-est included a total of 1,146,571 transcripts and an N50 of 1529 bp, with a value greater than 94% completeness for BUSCO evaluation in each queried database.

### Quality control of annotation

The transcriptome was functionally annotated by performing DIAMOND and InterProScan. By selecting the best hit for each database, the annotation matrix generated with DIAMOND has led to 153,048 and 95,942 contigs, and a total of 7,547 transcripts were annotated in at least one database.

InterProScan is a tool that combines different protein signature recognition methods of the InterPro member databases into one resource. It provides as result the corresponding InterPro accession numbers and, among other accession IDs, the GO and KEGG annotation.

## Data Availability

All the software programs used in this article (de-novo transcriptome assembly, pre- and post-assembly steps and transcriptome annotation) are listed with the version in the Methods paragraph. In case of no details on parameters the programs were used with the default settings.
